# Tissue Metabonomic Phenotyping for Diagnosis and Prognosis of Human Colorectal Cancer

**DOI:** 10.1038/srep20790

**Published:** 2016-02-15

**Authors:** Yuan Tian, Tangpeng Xu, Jia Huang, Limin Zhang, Shan Xu, Bin Xiong, Yulan Wang, Huiru Tang

**Affiliations:** 1 CAS Key Laboratory of Magnetic Resonance in Biological Systems, State Key Laboratory of Magnetic Resonance and Atomic and Molecular Physics, National Centre for Magnetic Resonance in Wuhan, Wuhan Institute of Physics and Mathematics, Chinese Academy of Sciences, Wuhan, 430071, China; 2 State Key Laboratory of Genetic Engineering, Collaborative Innovation Center for Genetics and Development, Ministry of Education Key Laboratory of Contemporary Anthropology, Metabonomics and Systems Biology Laboratory, School of Life Sciences, Fudan University, Shanghai, 200438, China; 3 Department of Oncology, Zhongnan Hospital of Wuhan University, Wuhan, 430071, China; 4 Department of Oncology, Renmin Hospital of Wuhan University, Wuhan, 430071, China; 5 Department of Hepatobiliary Surgery, China-Japan Friendship Hospital, Beijing, 100029, China; 6 Collaborative Innovation Center for Diagnosis and Treatment of Infectious Diseases, Hangzhou, 310058, China

## Abstract

Colorectal cancer (CRC) is one of the leading causes of cancer-related death worldwide and prognosis based on the conventional histological grading method for CRC remains poor. To better the situation, we analyzed the metabonomic signatures of 50 human CRC tissues and their adjacent non-involved tissues (ANIT) using high-resolution magic-angle spinning (HRMAS) ^1^H NMR spectroscopy together with the fatty acid compositions of these tissues using GC-FID/MS. We showed that tissue metabolic phenotypes not only discriminated CRC tissues from ANIT, but also distinguished low-grade tumor tissues (stages I-II) from the high-grade ones (stages III-IV) with high sensitivity and specificity in both cases. Metabonomic phenotypes of CRC tissues differed significantly from that of ANIT in energy metabolism, membrane biosynthesis and degradations, osmotic regulations together with the metabolism of proteins and nucleotides. Amongst all CRC tissues, the stage I tumors exhibited largest differentiations from ANIT. The combination of the differentiating metabolites showed outstanding collective power for differentiating cancer from ANIT and for distinguishing CRC tissues at different stages. These findings revealed details in the typical metabonomic phenotypes associated with CRC tissues nondestructively and demonstrated tissue metabonomic phenotyping as an important molecular pathology tool for diagnosis and prognosis of cancerous solid tumors.

Colorectal cancer (CRC) is one of the most prevalent cancers, causing high cancer-related mortality in both developed and developing countries^1^. According to the American Cancer Society, about 1.7 million new cancer cases and ~600,000 deaths from cancer are projected to occur in the United States in 2015, among which ~100,000 new cases and ~50,000 deaths will be from CRC^2^. In China, CRC mortality rapidly increased to become the fifth most common cancer-related deaths in 2012, and continued to rise^3^. Whilst proper prognosis for CRC is the key for reducing mortality rates, outstanding advances in early diagnosis and surgical treatment of CRC are required to improve the prognosis of CRC. Prognostic and improved treatment strategies are determined largely by the stages of cancer, therefore determining the stages of CRC is vital in the prevention of CRC-related mortality.

The current “gold standard” for CRC diagnosis is based on the colonoscopy in combined with histopathological examination whilst the most commonly accepted method for staging is based on Tumor Node Metastasis (TNM) or the Duke staging system^4^. These strategies involve detecting the depth of tumor invasion, the extension of lymphatic metastasis and distant metastasis microscopically. Although pathological TNM stage is a common predictive factor of predicting the prognosis and for planning treatments of CRC patients, heterogeneity of prognosis still exists in the same stage. Therefore, new, robust and reliable diagnostic approaches are urgently needed to improve the existing screening strategies.

Some new noninvasive CRC detection methods are being developed especially stool DNA (sDNA)^5^ and microRNA (miRNA) testing^6^. Four methylated genes including, a mutant form of *KRAS*, can be detected by the sDNA test which identifies 85% of patients with CRC and 54% of patients with adenomas, with 90% specificity^5^. Fecal miRNAs can be easily detected from both CRC patients and healthy subjects^6^ which have been used in the clinic for the noninvasive detection of CRC. Recently, proteomic^7^,^8^,^9^,^10^ and genomic^11^,^12^,^13^ studies have provided some more insights into the molecular phenotypes of CRC. Several genomic studies have found a number of genes responsible for inherited colorectal cancers including mutations in *APC* (Familial Adenomatous Polyposis), *hMSH2, hMLH1* (Lynch Syndrome), *MYH* (MYH polyposis), and *STK11* (Peutz-Jegher Syndrome)^14^,^15^,^16^. Proteomic studies have shown upregulations of glyceraldehyde-3-phosphate dehydrogenase (GAPDH) and malate dehydrogenase (MDH) but down-regulations of phosphoenolpyruvate carboxykinase (PEPCK), UDP-glucose pyrophosphorylase 2 (UGP2) and aconitate hydratase in CRC tissues compared to these in adjacent normal mucosa indicating the CRC-associated alterations in multiple metabolic pathways such as glycolysis/gluconeogenesis, glucuronate pathway and tricarboxylic acid cycle^17^,^18^,^19^. While alterations at the genomic and proteomic levels reflect the changes of a biological process, metabonomics considers the interaction of these processes with environmental factors and provides consequential results related to the biological event. In particular, ^1^H high resolution magic-angle spinning (HRMAS) nuclear magnetic resonance (NMR) analysis of tissue metabolic profiles *ex vivo* has shown great potential for cancer research due to the nondestructive nature of this technique^20^,^21^,^22^. Recently, similar metabonomics approaches have also shown potential in cancer diagnosis and prognosis^23^,^24^,^25^,^26^,^27^,^28^. Clinical metabonomic studies based on urine^29^,^30^, serum^31^,^32^ and tissue^33^,^34^,^35^ of CRC patients have provided some potential biomarkers for CRC detection and prognosis^36^,^37^. Despite these advances, there are still few studies on how tumor tissue metabonomic phenotypes correlate with the CRC staging especially in a molecular pathology context. Such information would be vital for understanding the processes of tumorigenesis, CRC grading, and hence CRC-related mortality reduction.

In this study, we used HRMAS NMR and gas chromatography-mass spectrometer (GC-MS) in combination with multivariate data analysis, to elucidate the metabonomic features of human CRC tissues at different stages, as well as their corresponding adjacent non-involved tissues (ANIT). The aims of this investigation are to define the tissue metabonomic characteristics associated with CRC at different stages and to explore the potentials of these molecular phenotypic profiles for diagnosis and prognosis of human colorectal cancer.

## Results

### ^1^H HRMAS NMR spectra of tissue samples

**Figure 1:**
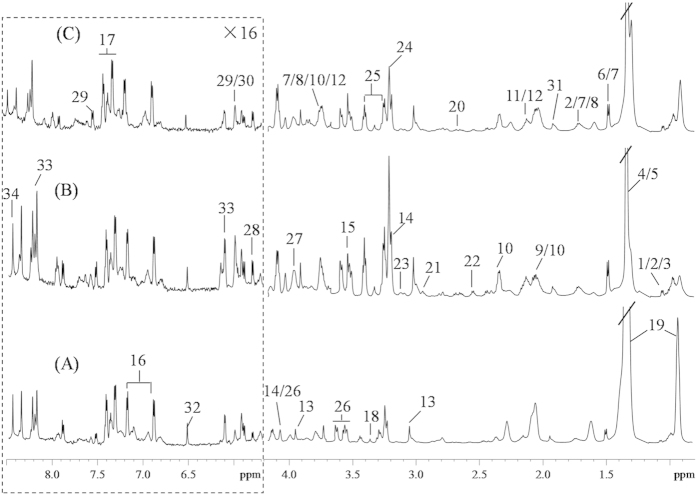
Average 600 MHz ^1^H HRMAS NMR spectra of ANIT (**A**), stage I CRC tumor (**B**) and stage IV CRC tumor (**C**). The region of *δ* 5.7–8.5 was vertically expanded 16 times compared with *δ* 0.8–4.2. Metabolite keys: 1, isoleucine; 2, leucine; 3, valine; 4, lactate; 5, threonine; 6, alanine; 7, lysine; 8, arginine; 9, proline; 10, glutamate; 11, methionine; 12, glutamine; 13, creatine; 14, choline; 15, glycine; 16, tyrosine; 17, phenylalanine; 18, *scyllo*-inositol; 19, lipid; 20, aspartate; 21, asparagine; 22, glutathione; 23, cysteine; 24, phosphorylcholine/glycerophosphocholine; 25, taurine; 26, *myo*-inositol; 27, phosphoethanolamine; 28, uracil; 29, cytosine; 30, isocytosine; 31, acetate; 32, fumarate; 33, inosine; 34, formate.

The average ^1^H NMR spectra of both CRC and ANIT samples (Fig. 1) showed a number of metabolites which were unambiguously assigned (Table S1) based on the literature data^34^,^35^ and further confirmed by a series of 2D NMR spectral data. The spectra of colonic tissues contained visible resonances from lipids, organic acids, amino acids and metabolites from choline and nucleosides (Table S1). Visual inspection of the spectra of these tissues revealed that the levels of some metabolites such as lipids, amino acids, and choline were obviously different between tumor tissues and ANIT (Fig. 1).

### Metabonomic characteristics of tumor tissues

**Figure 2:**
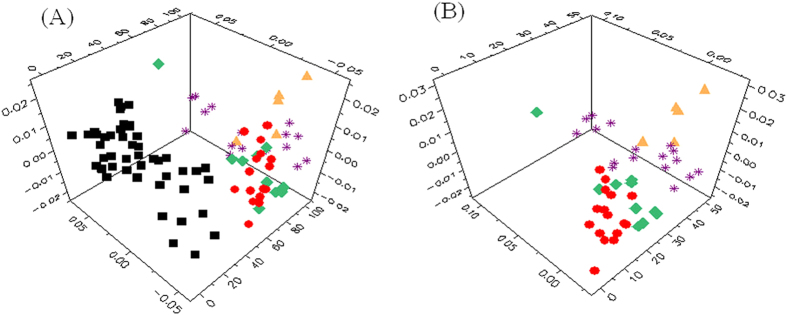
PCA scores plots obtained from NMR data of CRC tumor tissues at different stages (I–IV) with (**A**) or without (**B**) ANIT. ANIT (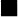), stage I (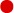), stage II (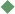), stage III (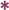), and stage IV (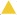).

**Figure 3:**
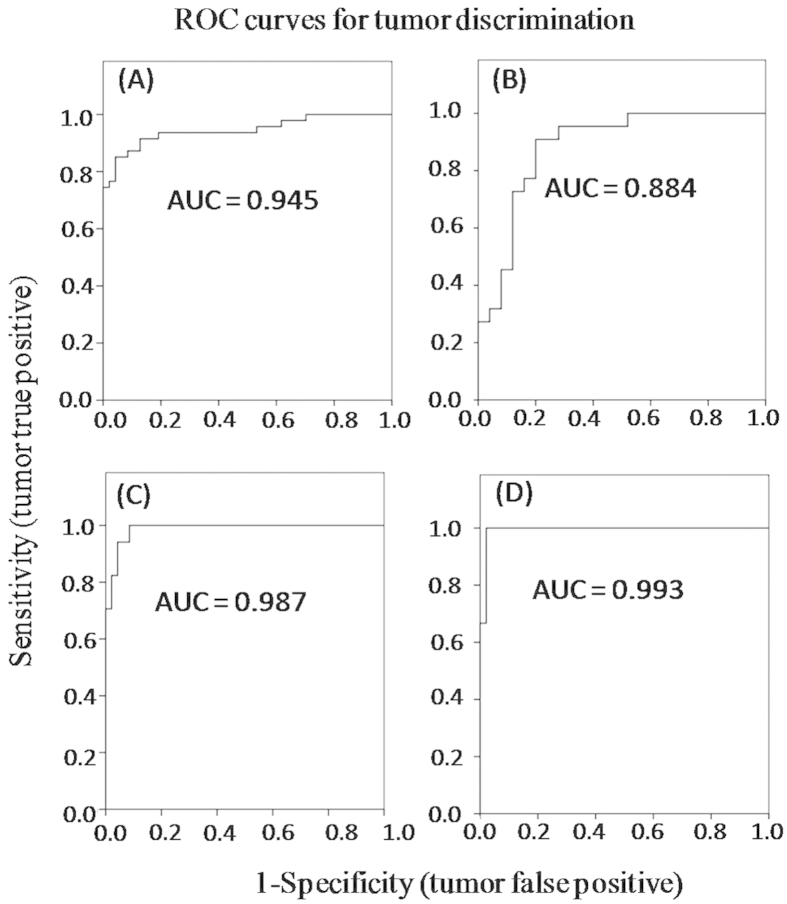
ROC curves determined using the cross-validated predicted Y-values of the ^1^H NMR OPLS-DA models from CRC tumor and ANIT. (**A**) ANIT *vs* CRC tumor, (**B**) stages I-II tumor *vs* stages III-IV tumor, (**C**) stage I tumor *vs* ANIT, (**D**) stage II tumor *vs* ANIT.

Principal component analysis (PCA) was conducted on the mean-centered ^1^H HRMAS NMR data from 50 pairs of CRC tumor and ANIT samples to generate an overview of the dataset and detect possible outliers. A clear indication of separation was observable between ANIT and CRC tumor tissues (Fig. 2A). Using orthogonal project to latent structure-discriminant analysis (OPLS-DA), we further analyzed the metabonomic profiles of CRC tumors at four different stages (I-IV). The results showed that stage I tumors were not distinguishable from stage II ones (Q^2^ = −0.36, *p* = 1), nor were stage III tumor tissues from stage IV ones (Q^2^ = 0.01, *p* = 1), though the limited number of samples from stage IV could be one potential reason for this observation (Fig. 2B and Figure S1). We then pooled the data together to consider them as low-grade tumor tissues (stages I-II) and high-grade one (stages III-IV) in the subsequent analyses. Receiver operating characteristic (ROC) analysis further confirmed clear differentiations between stage I tumor and ANIT as well as between stage II tumor and ANIT. Low-grade CRC tumors (stages I-II) were also successfully distinguished from the high-grade ones (stages III-IV) (Fig. 3).

**Figure 4:**
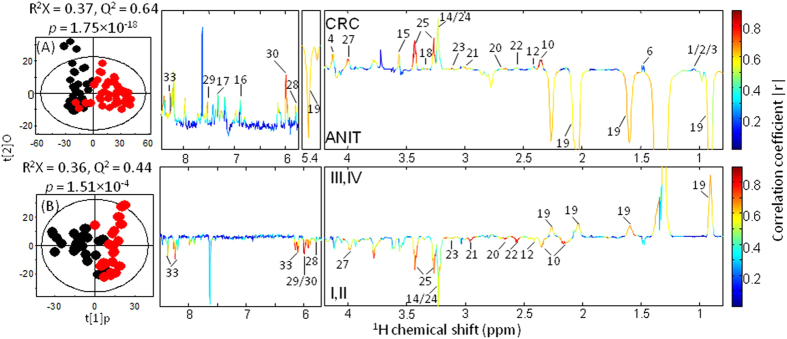
OPLS-DA scores (left) and coefficient-coded loadings plots (right) showing the discrimination between (**A**) ANIT (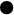) and CRC tumor (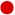) (n = 50, |*r*| > 0.29) and (**B**) stages I-II tumor (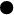) and stages III–IV tumor (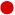) (n = 22, |*r*| > 0.41). Metabolite keys are given in Fig. 1 and Table S1.

OPLS-DA showed significant inter-group metabonomic differences between CRC tumors and ANIT, and between low-grade and high-grade CRC tumors (Fig. 4). Further evaluation using the CV-ANOVA method confirmed the statistical significance (*p* < 0.05) of these models (Fig. 4). Compared with ANIT, the CRC tissues contained relatively higher levels of lactate, choline, phosphorylcholine (PC), glycerophosphocholine (GPC), phosphoethanolamine (PE), *scyllo*-inositol, glutathione (GSH), taurine, uracil, cytosine, isocytosine, inosine and a range of amino acids, but lower levels of lipids (Fig. 4A, Table 1). Interestingly, when these 24 metabolites having significant differences between ANIT and CRC tissues were employed for inter-group differentiations, the resulting ROC model showed a good diagnostic power with an area under curve (AUC) of 0.965 (Figure S2A). Furthermore, the high-grade (stages III-IV) tumor tissues contained higher levels of lipids but lower levels of choline, PC, GPC, PE, GSH, taurine, uracil, isocytosine, inosine and some amino acids (glutamine, glutamate, aspartate, asparagine, glycine and cysteine) than the low-grade (stages I-II) tumor tissues (Fig. 4B, Table 1). Of note, the ROC curve generated from 15 significantly differentiated metabolites showed an AUC of 0.904 for distinguishing the low-grade tumors from the high-grade tumors (Figure S2B).

**Figure 5:**
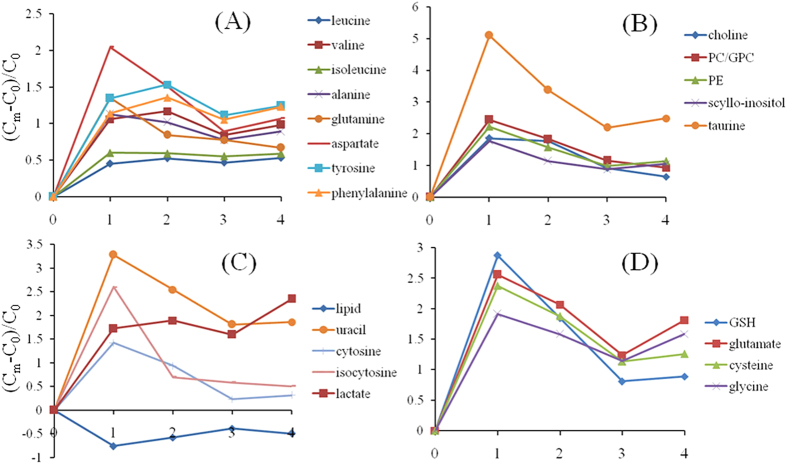
The ratios of metabolite changes for CRC tumor tissues at different stages (I-IV) against ANIT.

In order to obtain metabonomic phenotypes associated with CRC at various stages, tumor samples of each stage (I-IV) were compared with their corresponding ANIT using the OPLS-DA strategy (Figure S3) with the differentiated metabolites from the different pathological stages identified (Fig. 5). Amongst all these CRC tissues, stage I tumors exhibited the largest differences from their corresponding ANIT samples in terms of their metabonomic phenotypes. This was signified by the higher levels of choline, PC, GPC, PE, *scyllo*-inositol, taurine, uracil, cytosine, isocytosine, GSH and most of amino acids but lower levels of lipids in these tumor tissues. Such differences became less for the high-grade samples (stages III-IV) with exception of lactate, whose level continued to increase in the higher grade tumor tissues (Fig. 5A–D).

### Fatty acid compositions in the CRC-related tissue samples

**Figure 6:**
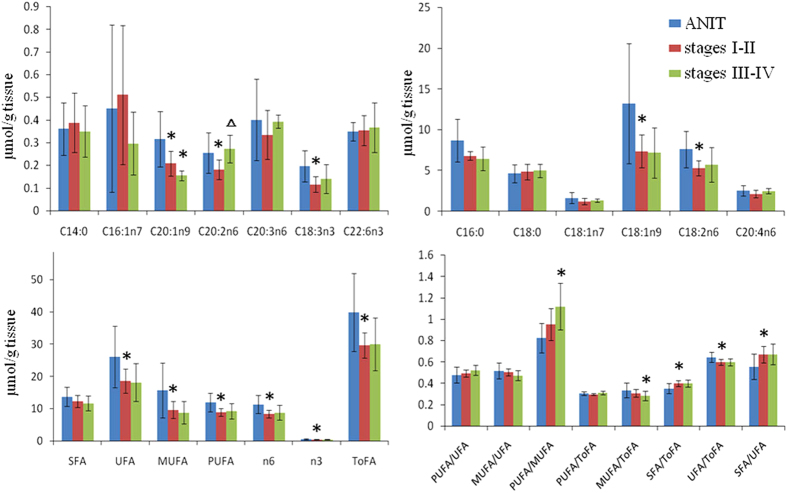
Fatty acid levels in ANIT and CRC tumor tissues. *p < 0.05 when compared to the ANIT, Δ p < 0.05 when compared to low-grade (stages I-II) tumor tissues.

To obtain the detailed information about these lipids showing inter-group differences, we analyzed the fatty acid composition in these tissues using GC-FID/MS. The low-grade tumor tissues had significantly lower levels of C18:1n9, C20:1n9, C18:2n6, C20:2n6, C18:3n3 than ANIT (Fig. 6 and Table S2). The levels for unsaturated fatty acids (UFA), monounsaturated fatty acids (MUFA), polyunsaturated fatty acids (PUFA) and total fatty acids (ToFA) were also lower than in ANIT (Fig. 6 and Table S2). In the higher-grade tumors (stages III-IV), only C20:1n9 level was significantly lower than that in the corresponding ANIT samples. Furthermore, the high-grade tumor tissues contained higher C20:2n6 level than the low-grade ones.

## Discussion

Prognostic treatment strategies largely depend on the stage of cancer and thus developing new screening methods with high sensitivity and specificity is critical for the early diagnosis of CRC. Most cancers have a long asymptomatic period and are difficult to detect during the early stages of cancer, few methods are able to detect the molecular events that underlie the initiation and progression of tumors. Pathological TNM stage has limitations and shortfalls in its own way since such approaches are at best confirmative rather than early discovering. Recently, an intriguing technology, called “intelligent knife” (iKnife), has been developed for clinical application, which detects the subtle differences of smoke between cancerous and healthy tissue generated from surgical knife within seconds in the surgery^38^. In order to obtaining information on cancer development and progression, in this study, we employed a holistic metabonomics approach to investigate the detailed metabolic phenotypes of CRC tissues at different stages, together with their corresponding ANIT. Based on their metabonomic phenotypic features, we have successfully discriminated CRC tumors, especially low-grade ones, from their ANIT, and distinguished the low-grade tumors from the high-grade ones with high specificity and sensitivity in both cases. Our data also showed that detailed metabolic compositions in tumors differed significantly from their corresponding ANIT, with the greatest differences observed between the low-grade tumors and their corresponding ANIT.

The differences in energy metabolisms were clearly observable between the CRC tissues and ANIT, with an significant increase in glycolytic capacity in the CRC tissues as compared with ANIT. Tumor cells are typified with “Warburg effect” by maintaining high aerobic glycolytic rates and high levels of glucose uptake together with lactate production^39^. Significantly higher alanine concentration in CRC tumors occurs during glucose utilization showing high glycolytic rates related to tumor malignancy^40^. Recent studies have also shown that the conversion of pyruvate to alanine occurs predominantly in precancerous tissues prior to observable morphologic or histological changes^41^. The alteration of lipid metabolism has also been observed in CRC tissues with enhanced lipogenesis as one of the most important feature in tumor tissues^42^. However, recent studies have found that tumor tissues can utilize both lipogenic and lipolytic pathways to acquire fatty acids for tumor cell proliferation^43^.

In our studies, a significantly lower level of lipids indicated that lipolysis and fatty acid oxidation are the dominant bioenergetic pathways in CRC tissues. In addition, we also detected altered fatty acid composition in the CRC tumor tissues. Significantly lower levels of n3- and n6-type PUFA were observed in CRC tumor tissues as compared with ANIT, suggesting an association of inflammation with CRC. Association of lower levels of PUFAs in CRC tumor could be attributed to the pro-tumor genesis of cyclooxygenase 2 (COX-2), an enzyme converting PUFAs to prostaglandins during inflammatory and tumorigenic reactions^44^. In our study, we also found higher levels of a range of amino acids in the CRC tumors as compared with ANIT. A growing tumor needs, more so than any normal tissues, a good supply of energy. Many of these amino acids can enter the tricarboxylic acid cycle (TCA) to provide energy for fast tumor growth, particularly when anaerobic metabolism is inefficient. Compared with ANIT, we further observed higher levels of cysteine and GSH in CRC tumors, which has also been observed in human esophageal cancer^45^. The rate-limiting precursor for GSH synthesis, cysteine, was present in higher levels concurrently with GSH in CRC tissues than in ANIT indicating a redox status shift in the CRC tumor tissues. GSH is essential for cell growth and therefore low levels of GSH promotes apoptosis, whereas high levels of GSH have been associated with resistance to chemotherapy^46^.

We further observed higher levels of nucleotides, nucleosides and nucleobases in CRC tissues as compared with ANIT. These metabolites are key components of DNA and RNA structures and their biosynthetic dysregulations have some profound effects on cellular physiology, which can lead to neoplastic transformation of cells^47^. Higher uracil level in CRC tumors than in ANIT observed in our study has also been reported previously in CRC and hepatocellular carcinoma due to reduced dihydropyrimidine dehydrogenase (DPD) activity^48^,^49^. Down-regulation of DPD expression may therefore create a favorable environment for tumor cell proliferation, leading to decrease uracil catabolism^50^,^51^. Furthermore, up-regulation of cytosine and its isomer isocytosine in CRC tumors found in our study has been observed previously in leukaemia^52^.

Higher levels of choline metabolites such as choline, PC, GPC and PE observed here in CRC tissues than in ANIT have been reported in other malignant tumors^35^,^53^,^54^. This is consistent with high activity of both biosynthetic (choline kinase) and catabolic (phosphatidylcholine-PLC/PLD) enzymes observed in ovarian carcinoma contributing to the observed choline-containing compounds accumulation in CRC tumor^55^. Our observation of the higher levels of taurine and *scyllo*-inositol in CRC tissues than in ANIT was indicative of a localized change in osmotic regulation in CRC tumor tissues since both these metabolites may function as osmotic regulative metabolites^35^ though taurine could have multiple functions.

In summary, tissue metabonomic analyses using the combined HRMAS and GC-FID/MS techniques have revealed significant differences in terms of metabolic phenotypes between 50 human CRC tumor tissues and their corresponding adjacent non-involved tissues. Amongst all CRC tissues, the stage I CRC tumor tissues showed greatest metabonomic differences from their corresponding adjacent non-involved tissues. Using the same approach, such differences were also readily observable between the low-grade (stage I-II) and high grade (stage III-IV) CRC tissues. The differentiated metabolites involved in energy metabolism (glycolysis), osmotic regulations, membrane biosynthesis/degradations together with metabolisms of proteins and nucleotides. The combination of these significantly differentiated metabolites were powerful molecular phenotypic features for differentiating CRC and adjacent non-involved tissues as well as for distinguishing low-grade and high grade CRC. These findings provided crucial details for insights into CRC biology and demonstrated tissue metabonomic phenotyping as a potentially important molecular pathological approaches for diagnosis and prognosis of solid tumors.

## Materials and Methods

### Ethics statement

This study was approved by the local ethic committee of Zhongnan Hospital of Wuhan University with an informed consent form signed by all participants. All experimental protocols were in accordance with the approved guidelines for safety requirements of Wuhan Institute of Physics and Mathematics, University of Chinese Academy of Sciences.

### Chemicals

Deuterium oxide (D_2_O, 99.9% D) was obtained from Cambridge Isotope Laboratories, Inc. (Miami, USA.). Methanol, hexane, and K_2_CO_3_ were obtained all in analytical grade from Sinopharm Chemical Reagent Co. Ltd. (Shanghai, China). Methyl heptadecanoate, methyl tricosanate, and acetyl chloride (99.0%) were purchased from Sigma-Aldrich (St. Louis, MO) whereas 3,5-Di-tert-butyl–4-hydroxytoluene (BHT) and a mixed standard methyl esters of 37 fatty acids were obtained from Supelco (Bellefonte, PA).

### Clinical sample collection

CRC tumors including 16 colon cancer and 34 rectal cancer together with the corresponding adjacent non-involved tissues (ANIT) were collected from 50 CRC patients (aged 42–70 years) at different stages (Table 2). ANIT samples were taken at least 5–10 cm away from the edges of the tumor. Both CRC and ANIT were diagnosed with clinical histopathological approaches at the Department of Pathology, Zhongnan Hospital of Wuhan University, and cancer stages were determined according to TNM classification. All tissues were snap-frozen in liquid nitrogen after resection at surgery and stored at −80 °C until further analysis.

### ^1^H HRMAS NMR spectroscopic analysis

All HRMAS NMR experiments were carried out at 283 K on a Bruker AVIII 600 MHz spectrometer (Bruker Biospin, Germany) using a triple-resonance HRMAS probe with a sample spin rate of 5000 Hz. Each tissue sample (about 15 mg) was individually placed in D_2_O saline and inserted into a 4 mm diameter zirconium oxide rotor for all NMR acquisitions. In order to attenuate NMR signals of macromolecules, a Carr-Purcell-Meiboom-Gil (CPMG) spin-echo spectrum was collected for each sample with the spin-spin relaxation delay (2nτ) set to 70 ms for all samples. The 90° pulse length was adjusted to about 10 μs for each sample and a total of 128 transients were collected into 32k data points with a spectral width of 20 ppm.

To facilitate assignments, a series of 2D NMR spectra were acquired and processed as described previously^56^,^57^ for some selected samples including ^1^H-^1^H correlation spectroscopy (COSY), ^1^H-^1^H total correlation spectroscopy (TOCSY), ^1^H J-resolved spectroscopy (JRES), ^1^H−^13^C heteronuclear single quantum correlation spectroscopy (HSQC), and ^1^H−^13^C heteronuclear multiple bond correlation spectroscopy (HMBC). For all spectral acquisitions, water signal was suppressed with a weak continuous wave irradiation during recycle delay.

### Spectral processing and multivariate statistical data analysis

For all one-dimensional spectra, an exponential window function was applied with a line-broadening factor of 1.0 Hz prior to Fourier transformation. All NMR spectra were then phase- and baseline-corrected manually using Topspin (V3.0, Bruker Biospin, Germany). The chemical shifts of spectra were all referenced to the methyl protons of alanine (*δ*1.48). The spectral ranges of *δ* 0.50–8.50 were divided into bins with an equal width of 0.004 ppm (2.4 Hz) using AMIX software package (V3.9.5, Bruker Biospin, Germany). The residual water signal in the regions of *δ* 4.20–5.20 was discarded prior to data analyses together with ethanol resonances (*δ* 1.14–1.18, *δ* 3.62–3.70), which were probably introduced during sample collections. The bucketed spectral data were normalized to the sum of total integrals of each spectrum.

The datasets were then imported into SIMCA-P+ (V12.0, Umea, Sweden) for multivariate data analyses. PCA was carried out with the mean-centered data and the scores plots were employed to visualize group clusterings and to detect possible outliers. OPLS-DA was further conducted with a 7-fold cross-validation approach by using the unit-variance scaled data using NMR data as the *X*-matrix and group information as the *Y*-matrix^58^. The quality of these models was described by R^2^X representing the explained variations and Q^2^Y indicating the model predictability. CV-ANOVA methods were employed to assess the robustness of the model (to the level of *p* < 0.05)^59^. The back-transformed loadings were plotted with the correlation coefficients of metabolites color-coded using an in-house developed script to show these variables (or metabolites) contributed to the intergroup separations. The ratios of metabolite changes for CRC tumor tissues at different stages were also calculated against the corresponding ANIT in the form of [C_m_–C_0_]/C_0_, where C_m_ and C_0_ stood for the peak areas of a particular metabolite signal (having least overlapping) in tumor tissues and ANIT, respectively.

### Receiver Operating Characteristic (ROC) Curve

ROC curves were obtained from the Y-predicted values to evaluate the predicative ability of OPLS-DA models. AUC was computed using the performance curve algorithm from SPSS 18.0 (SPSS Inc., Chicago, IL, USA).

### GC-FID/MS analysis of tissue fatty acid composition

Tissue fatty acids were measured using a previously reported method^60^ with some minor modifications. Each 15 mg tissue sample was homogenized individually in cold methanol using a TissueLyser (20 Hz, 90 s). After acetylchloride catalyzed methylation^60^, methyl esters of all fatty acids were separated, identified and quantified on a Shimadzu GCMS-QP2010Plus spectrometer (Shimadzu Scientific Instruments, USA) equipped with a GC system, a mass spectrometer with an EI source and a flame ionization detector (FID). An Agilent DB-225 capillary GC column (10 m, 0.1 mm ID, 0.1 μm film thickness) was employed with helium gas as carrier and makeup gas. Sample injection volume was 1 μL with a splitter (1:60). The GC and detection parameters were set as previously reported^60^. Methylated fatty acids were identified by comparing with a mixture of known standards and confirmed with their mass spectral data from standard libraries. The results were expressed as μmol fatty acids per gram tissue.

## Additional Information

**How to cite this article**: Tian, Y. *et al.* Tissue Metabonomic Phenotyping for Diagnosis and Prognosis of Human Colorectal Cancer. *Sci. Rep.* **6**, 20790; doi: 10.1038/srep20790 (2016).

## Supplementary Material

Supplementary Information

## Figures and Tables

**Table 1:** Correlation Coefficients for Metabolites having significantly differences between CRC tumors and ANIT, and between stages III-IV and stages I-II tumors.

Metabolite (no.)	CRC *vs* ANIT	stages III-IV *vs* stages I-II
Lipid (19)	−0.62	0.62
Lactate (4)	0.58	—
Leucine (2)	0.44	—
Valine (3)	0.41	—
Isoleucine (1)	0.39	—
Alanine (6)	0.32	—
Glutamine (12)	0.32	−0.50
Glutamate (10)	0.89	−0.65
Aspartate (20)	0.54	−0.55
Aspargine (21)	0.51	−0.80
Cysteine (23)	0.49	−0.53
Glycine (15)	0.60	−0.47
Tyrosine (16)	0.46	—
Phenylalanine (17)	0.43	—
Choline (14)	0.37	−0.46
PC/GPC (24)	0.37	−0.50
PE (27)	0.76	−0.65
*Scyllo*-inositol (18)	0.61	—
GSH (22)	0.54	−0.75
Taurine (25)	0.79	−0.67
Uracil (28)	0.61	−0.75
Cytosine (29)	0.62	—
Isocytosine (30)	0.76	−0.63
Inosine (33)	0.60	−0.62

The coefficients were from OPLS-DA results; positive and negative signs indicate positive and negative correlations, respectively.

**Table 2:** Clinical information of CRC patients.

	patients for HRMAS NMR	patients for GC-MS
Number	50	16
Age (median, range)	56, 42–70	58, 45–64
Male/female ratio	30/20	11/5
Stage I	16	6
Stage II	12	4
Stage III	17	3
Stage IV	5	3
colon cancer	16	6
rectal cancer	34	10
