# Supplementary material for: Tissue Metabonomic Phenotyping for Diagnosis and Prognosis of Human Colorectal Cancer
Source: Sci Rep. 2016 Feb 15;6:20790. doi: 10.1038/srep20790 (PMC4753490; doi:10.1038/srep20790)
Supplement: Supplementary Information [file srep20790-s1.pdf]

Tissue Metabonomic Phenotyping for Diagnosis and Prognosis of Human Colorectal Cancer

Yuan Tian[†ζ], Tangpeng Xu[‖‡ζ], Jia Huang[‖⊥], Limin Zhang[†], Shan Xu[†], Bin Xiong[‖*], Yulan Wang[†#*], Huiru Tang[†Φ*]

[†]CAS Key Laboratory of Magnetic Resonance in Biological Systems, State Key Laboratory of Magnetic Resonance and Atomic and Molecular Physics, National Centre for Magnetic Resonance in Wuhan, Wuhan Institute of Physics and Mathematics, Chinese Academy of Sciences, Wuhan, 430071, China

[Φ]State Key Laboratory of Genetic Engineering, Collaborative Innovation Center for Genetics and Development, Metabolomics and Systems Biology Laboratory, School of Life Sciences, Fudan University, Shanghai, 200438, China

[‖]Department of Oncology, Zhongnan Hospital of Wuhan University, Wuhan, 430071, China

[‡]Department of Oncology, Renmin Hospital of Wuhan University, Wuhan, 430071, China

[⊥]Department of Hepatobiliary Surgery, China-Japan Friendship Hospital, Beijing, 100029，China

[#]Collaborative Innovation Center for Diagnosis and Treatment of Infectious Diseases, Hangzhou, 310058, China

Supplementary data：

Figure S1. PCA (left) and OPLS-DA (right) scores plots obtained from NMR data of different pathological stages (I-IV) of CRC tumor tissues. (A) stage I *vs* stage II (OPLS-DA: R^2X = 0.17, Q^2 = -0.36, p = 1), (B) stage II *vs* stage III (OPLS-DA: R^2X = 0.38, Q^2 = 0.32, p = 0.006), and (C) stage III *vs* stage IV (OPLS-DA: R^2X = 0.47, Q^2 = 0.01, p = 1).

Figure S2. ROC curve determined using all metabolites that have (A) |r| > 0.29 in comparison of ANIT and CRC tumor, (B) |r| > 0.41 in comparison of stages I-II tumor and stages III-IV tumor.

Figure S3. OPLS-DA scores (left) and coefficient plots (right) showing the discrimination between different pathological stages (I-IV) of CRC tumor and ANIT. (A) stage I *vs* ANIT (R^2X = 0.41, Q^2 = 0.59, $p = 3.49\times10^{-7}$), (B) stage II *vs* ANIT (R^2X = 0.43, Q^2 = 0.46, $p = 1.17\times10^{-2}$), (C) stage III *vs* ANIT (R^2X = 0.43, Q^2 = 0.56, $p = 3.11\times10^{-3}$), and (D) stage IV *vs* ANIT (R^2X = 0.58, Q^2 = 0.65, $p = 1.09\times10^{-2}$). Keys to metabolites assignment are given in Table S1.

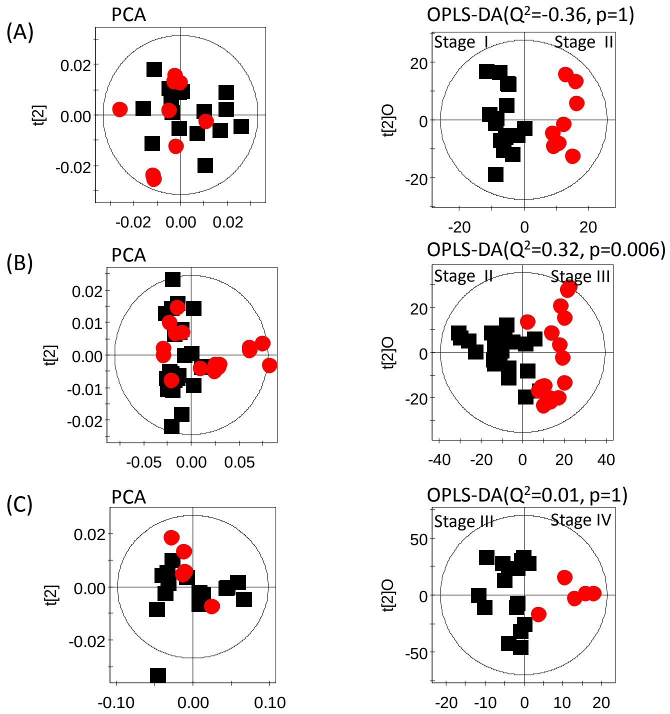

Figure S1. PCA (left) and OPLS-DA (right) scores plots obtained from NMR data of different pathological stages (I-IV) of CRC tumor tissues. (A) stage I *vs* stage II (OPLS-DA: $R^2X = 0.17$, $Q^2 = -0.36$, $p = 1$), (B) stage II *vs* stage III (OPLS-DA: $R^2X = 0.38$, $Q^2 = 0.32$, $p = 0.006$), and (C) stage III *vs* stage IV (OPLS-DA: $R^2X = 0.47$, $Q^2 = 0.01$, $p = 1$).

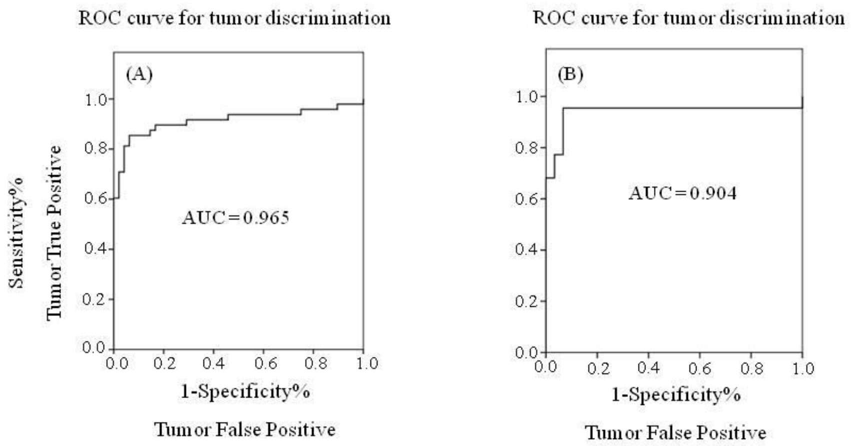

Figure S2. ROC curves determined using all metabolites having significant inter-group differences (A) ANIT and CRC tumor, (B) Stages I-II tumor and stages III-IV tumor.

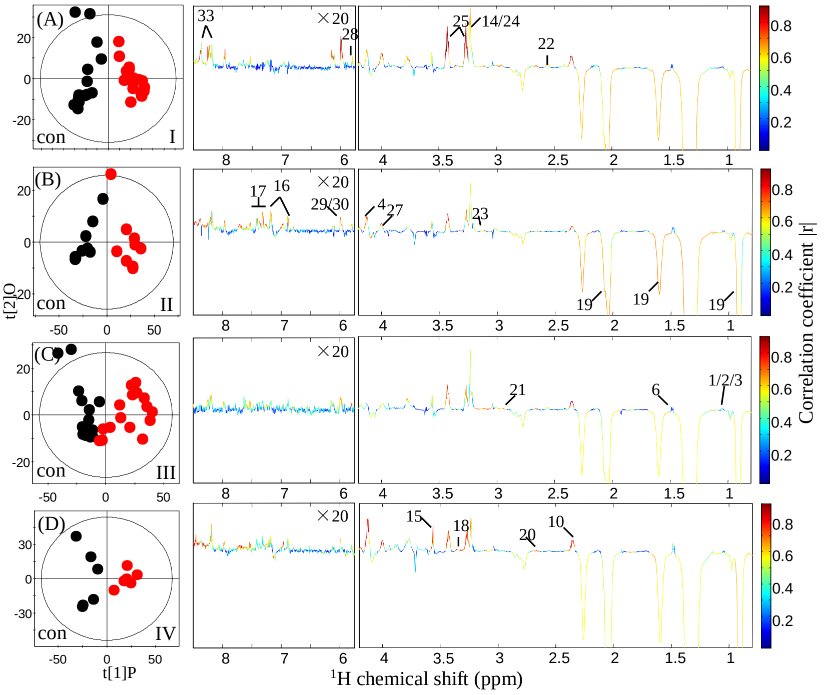

Figure S3. OPLS-DA scores (left) and coefficient plots (right) showing the discrimination between different pathological stages (I-IV) of CRC tumor and ANIT (con). (A) stage I *vs* ANIT (R^2X = 0.41, Q^2 = 0.59, p = 3.49×10^{-7}), (B) stage II *vs* ANIT (R^2X = 0.43, Q^2 = 0.46, p = 1.17×10^{-2}), (C) stage III *vs* ANIT (R^2X = 0.43, Q^2 = 0.56, p = 3.11×10^{-3}), and (D) stage IV *vs* ANIT (R^2X = 0.58, Q^2 = 0.65, p = 1.09×10^{-2}). Keys to metabolites assignment are given in Table S1.

Table S1. Resonance assignments metabolites found in CRC and adjacent non-involved tissues.

NO.	Metabolites	Moieties	δ ^{1}H (ppm) and multiplicity [a]
1	isoleucine	$\delta CH_3,\gamma CH_3,\gamma CH_2,\gamma' CH_2,\beta CH,\alpha CH$	0.94(t),1.01(d),1.25(m),1.48(m),1.98(m),3.67(d)
2	leucine	$\delta CH_3,\delta CH_3,\gamma CH,\beta CH_2,\alpha CH$	0.96(d),0.97(d),1.69(m),1.71(m),3.74(t)
3	valine	$\gamma CH_3,\gamma CH_3,\beta CH,\alpha CH$	0.99(d),1.04(d),2.27(m),3.62(d)
4	lactate	$\beta CH_3,\alpha CH$	1.33(d),4.11(q)
5	threonine	$\gamma CH_3,\alpha CH,\beta CH$	1.33(d),3.59(d),4.26(m)
6	alanine	$\beta CH_3,\alpha CH$	1.48(d),3.79(q)
7	lysine	$\gamma CH_2,\delta CH_2,\beta CH_2,\varepsilon CH_2,\alpha CH$	1.48(m),1.72(m),1.90(m),3.03(t),3.76(t)
8	arginine	$\gamma CH_2,\beta CH_2,\delta CH_2,\alpha CH$	1.73(m),1.93(m),3.03(t),3.75(t)
9	proline	$\gamma CH_2,\beta CH_2,\beta' CH_2,\delta CH_2,\delta' CH_2,\alpha CH$	2.01(m),2.07(m),2.36(m),3.34(m),3.45(m),4.13(m)
10	glutamate	$\beta CH_2,\gamma CH_2,\alpha CH$	2.10(m),2.36(m),3.77(m)
11	methionine	$\delta CH_3,\beta CH_2,\gamma CH_2,\alpha CH$	2.14(s),2.16(m),2.65(dd),3.86(m)
12	glutamine	$\beta CH_2,\gamma CH_2,\alpha CH$	2.15(m),2.46(m),3.77(m)
13	creatine	CH_3,CH_2	3.04(s),3.93(s)
14	choline	$N(CH_3)_3,NCH_2,OCH_2$	3.21(s),3.52(m),4.07(m)
15	glycine	CH_2	3.57(s)
16	tyrosine	βCH2,β'CH2,αCH,3 or 5CH,4 or 6CH	3.06(dd),3.20(dd),3.94(dd),6.91(d),7.20(d)
17	phenylalanine	βCH2,β'CH2,αCH,2 or 6CH,4CH, 3 or 5CH	3.13(dd),3.29(dd),3.98(dd),7.33(m),7.38(m), 7.43(m)
18	*scyllo*-inositol	ring-CH	3.35(s)
19	lipid (fatty acids)	$CH_3(CH_2)_n,CH_3CH_2CH_2C{=},CH2,$ $CH_2C{=}C,{=}CCH_2C{=},HC{=}CH$	0.85(m),0.89(m),1.27(m),2.03(m),1.77(m),5.30(m)
20	aspartate	$\beta CH_2,\beta' CH_2,\alpha CH$	2.68(m),2.82(m),3.91(m)
21	asparagine	$\beta CH_2,\beta' CH_2,\alpha CH$	2.86(dd),2.96(dd),4.00(m)
22	glutathione(GSH)	$CH_2,O{=}CCH_2,SHCH_2,NH_2CH,O{=}CCH$	2.10(m),2.55(m),2.98(m),3.77(m),4.56(m)
23	cysteine	$CH_2,'CH_2,CH$	3.04(m),3.08(m),3.98(m),
24	phosphorylcholine(PC)	CH_3,NCH_2,OCH_2	3.22(s),3.59(t),4.25(t)
	glycerophosphocholine(GPC)	CH_3,OCH_2	3.22(s),4.12(t)
25	taurine	CH_2SO_3,NCH_2	3.25(t),3.43(t)
26	*myo*-inositol	5CH,1 or 3CH,2 or 4CH	3.27(t),3.54(m),3.62(m),4.05(t)
27	phosphoethanolamine(PE)	NCH_2,OCH_2	3.22(m),3.99(m)
28	uracil	CH,CH	5.81(d),7.54(d)
29	cytosine	H6,H5	5.98(d),7.51(d)
30	isocytosine	H5,H6	5.99(d),7.62(d)
31	acetate	CH_3	1.92(s)
32	fumarate	CH	6.51(s)
33	inosine	$CH_2,'CH_2,5H',4H',2H',8H,2H$	3.85(dd),3.92(dd),4.28(q),4.44(t),6.10(d),8.24(s),8.34(s)
34	formate	CH	8.45(s)

[a] s, singlet; d, double; t, triplet; q, quartet; m, multiplet; dd, double of doubles.

Table S2. Fatty acid composition in tissues.

	ANIT[a] (n = 12)	low-grade (n = 10)	high-grade (n = 6)	p values (ANIT *vs* low-grade)	p values (ANIT *vs* high-grade)	p values (low-grade *vs* high-grade)
SFA[b]	13.752±2.919	12.339±1.931	11.772±2.333	2.36E-1	2.12E-1	6.33E-1
C14:0	0.361±0.115	0.388±0.131	0.350±0.113	6.51E-1	8.70E-1	6.02E-1
C16:0	8.678±2.640	6.800±0.539	6.436±1.456	5.33E-2	1.04E-1	5.27E-1
C18:0	4.647±1.100	4.842±0.555	4.986±0.781	6.86E-1	5.55E-1	7.81E-1
UFA[b]	26.096±9.511	18.597±3.681	18.167±5.799	3.98E-2	1.14E-1	8.67E-1
MUFA[b]	15.759±8.520	9.687±2.629	8.824±3.507	4.46E-2	1.06E-1	6.10E-1
C16:1n7	0.450±0.369	0.511±0.307	0.297±0.138	7.03E-1	3.91E-1	1.69E-1
C18:1n7	1.657±0.689	1.224±0.367	1.352±0.199	1.02E-1	2.08E-1	5.40E-1
C18:1n9	13.202±7.342	7.379±2.048	7.193±3.076	2.74E-2	1.05E-1	8.94E-1
C20:1n9	0.317±0.122	0.208±0.055	0.155±0.022	2.45E-2	1.33E-2	9.05E-2
PUFA[b]	12.000±2.831	8.910±1.207	9.343±2.379	8.09E-3	9.56E-2	6.55E-1
n6 [b]	11.384±2.747	8.423±1.218	8.836±2.260	8.87E-3	9.77E-2	6.59E-1
C18:2n6	7.601±2.222	5.320±0.921	5.712±2.145	1.68E-2	1.49E-1	7.15E-1
C20:2n6	0.255±0.089	0.181±0.043	0.272±0.061	4.66E-2	7.13E-1	8.73E-3
C20:3n6	0.401±0.180	0.335±0.108	0.393±0.029	3.97E-1	8.97E-1	2.18E-1
C20:4n6	2.526±0.628	2.123±0.450	2.488±0.336	1.31E-1	9.02E-1	1.42E-1
n3[b]	0.616±0.125	0.487±0.377	0.553±0.086	1.33E-2	3.77E-1	1.56E-1
C18:3n3	0.196±0.068	0.116±0.034	0.140±0.064	1.01E-2	1.66E-1	3.96E-1
C22:6n3	0.349±0.040	0.355±0.067	0.367±0.109	8.42E-1	7.42E-1	7.97E-1
ToFA[b]	39.848±12.079	29.598±3.885	29.939±8.119	2.78E-2	1.24E-1	9.20E-1
n6/n3[b]	18.635±3.485	17.593±3.566	17.496±1.491	5.29E-1	5.02E-1	9.55E-1
PUFA/UFA[b]	0.480±0.074	0.494±0.032	0.525±0.049	6.20E-1	2.44E-1	1.93E-1
MUFA/UFA[b]	0.520±0.074	0.506±0.032	0.475±0.049	6.20E-1	2.44E-1	1.93E-1
PUFA/MUFA[b]	0.826±0.140	0.952±0.150	1.123±0.218	9.52E-2	1.19E-2	1.07E-1
PUFA/ToFA[b]	0.306±0.017	0.299±0.008	0.314±0.015	3.06E-1	4.53E-1	1.00E-1
MUFA/ToFA[b]	0.339±0.070	0.309±0.034	0.286±0.044	2.69E-1	1.52E-2	1.56E-1
SFA/ToFA[b]	0.354±0.048	0.401±0.027	0.400±0.034	2.06E-2	7.91E-2	9.81E-1
UFA/ToFA[b]	0.646±0.048	0.599±0.027	0.600±0.034	2.06E-2	7.91E-2	9.81E-1
SFA/UFA[b]	0.557±0.119	0.672±0.076	0.672±0.096	2.38E-2	8.40E-2	9.78E-1

[a] data are presented as mean ± SD (μmol/g tissue)

[b] SFA: saturated fatty acids; UFA: unsaturated fatty acids; MUFA: monounsaturated fatty acids; PUFA: polyunsaturated fatty acids; n6: n6 PUFA ; n3: n3 PUFA ;ToFA: total fatty acids; n6/n3: n6-to-n3 ratio; PUFA/UFA: PUFA-to-UFA ratio; MUFA/UFA: MUFA-to-UFA ratio; PUFA/MUFA: PUFA-to-MUFA ratio; PUFA/ToFA: PUFA-to-ToFA ratio; MUFA/ToFA: MUFA-to-ToFA ratio; SFA/ToFA: SFA-to-ToFA ratio; UFA/ToFA: UFA-to-ToFA ratio; SFA/UFA: SFA-to-UFA ratio.
